# Fully-Metallic Additively Manufactured Monolithic Double-Ridged Waveguide Rotman Lens in the K/K_a_-Band

**DOI:** 10.3390/s23146573

**Published:** 2023-07-21

**Authors:** Nelson J. G. Fonseca, Sophie-Abigaël Gomanne, José Rico-Fernández, Petar Jankovic, Jaione Galdeano, Giovanni Toso, Piero Angeletti, Manuel Arrebola, Oscar Quevedo-Teruel

**Affiliations:** 1 European Space Agency, 2200AG Noordwijk, The Netherlands; sophie.gomanne@hanwha-phasor.com (S.-A.G.); petar.jankovic@aeroespacial.sener (P.J.); jaione.galdeano@esa.int (J.G.); giovanni.toso@esa.int (G.T.); piero.angeletti@esa.int (P.A.); 2 TheNextPangea SL, 33418 Asturias, Spain; pepe.rico@northern-waves.com; 3 Department of Electrical Engineering, Universidad de Oviedo, 33203 Gijón, Spain; arrebola@uniovi.es; 4 School of Electrical Engineering and Computer Science, KTH Royal Institute of Technology, 11428 Stockholm, Sweden; oscarqt@kth.se

**Keywords:** fully-metallic antenna, Rotman lens, multiple beam antenna, satellite communication, additive manufacturing

## Abstract

This paper reports on the design and experimental validation of a fully-metallic double-ridged waveguide 10 × 10 Rotman lens additively manufactured as a single part. The wide band operation of this quasi-optical beamformer enables us to cover the uplink and downlink frequencies allocated to satellite communications in the K/K${}_{a}$-band, from 17.3 GHz to 30 GHz. The feeding port design was adjusted to enable vertical printing, thus minimizing the use of supporting structures. A prototype was manufactured and tested. The reported results indicate losses in the range of 0.5 dB in the lower-frequency band and 0.8 dB in the upper-frequency band, including the waveguide transitions added for test purposes. The measured reflection and coupling coefficients remain below −11.5 dB over the operating band. The standard deviation of the residual phase error across the array ports is below 5° in simulation and below 10° in measurements. Array factors synthesized using the scattering parameters confirm the good stability of the beamforming functionality over the wide frequency band analyzed. This monolithic design is a promising step toward more integrated antenna systems, such as a compact dual-stack configuration for planar array design.

## 1. Introduction

Over the past decade, there has been an increased interest in quasi-optical antenna solutions with the development of new millimeter-wave applications, such as terrestrial and satellite wireless communications, as well as surveillance and automotive radar systems. Quasi-optical antenna solutions [1] are generally simpler than alternative high-gain antennas, such as arrays using circuit-type beamforming networks [2], and are thus more robust to manufacturing and assembly tolerances at higher frequencies. A wide variety of quasi-optical beamforming solutions has been reported in the literature. They may be categorized into three-dimensional (3-D) and two-dimensional (2-D) solutions, producing a planar aperture and a line source, respectively. Some solutions, such as the well-known Luneburg lens [3], may be designed as a 3-D [4,5] or a 2-D solution [6,7,8]. A variant, known as the Rinehart–Luneburg lens or geodesic lens [9], is a 2-D quasi-optical beamformer providing a continuous curved aperture and wide scanning range. More compact geodesic lens designs implementing a folded profile were reported recently [10,11]. Alternative PPW beamformers using mechanically simple ridges have also been proposed for space applications [12,13,14,15] and 5G communications [16].

Although slightly more complex in implementation due to the discretization of their aperture, constrained lenses [17] are better adapted to feed planar or linear arrays, enabling the integration of amplifiers at the element level to produce active antennas [18,19]. A particular case of interest is the Rotman lens [20], which may be used to feed a linear array, possibly extended into a planar array [21,22,23], achieving wide scanning in the beamforming plane ranging from about ±30° with a standard focal arc [24] to ±50° with an improved focal curve design [25,26]. The Rotman lens designs reported in the literature mostly use printed circuit board (PCB) technology, with substrate-integrated transmission lines [27]. Designs in stripline technology are reported in [28,29] for space applications. At millimeter-wave frequencies, waveguide technology is often preferred to avoid excessive insertion losses.

Few examples of Rotman lenses in waveguide technology are reported in the literature [30,31]. They use E-plane waveguides to reduce the spacing between adjacent ports to half-a-wavelength, with the drawback that the parallel plate waveguide (PPW) section operates in a dispersive transverse electric (TE) mode, thus limiting the operating bandwidth. More recently, a double-ridged waveguide Rotman lens design was proposed and manufactured in two mechanical parts using selective laser sintering [32]. This configuration provides a port spacing small enough to maintain good performance of the lens over a wider frequency band while enabling the PPW section to operate in a transverse electromagnetic (TEM) mode leading to a true time delay design with better overall RF performance [33]. A ridge-gap waveguide design has been reported at 60 GHz, with similar benefits [34]. A double-ridged waveguide design has also been proposed by simultaneously covering the uplink and downlink frequencies allocated to satellite communications in the K/K${}_{a}$-band [35].

This work reports on a similar wideband double-ridged waveguide Rotman lens design in the K/K${}_{a}$-band adapted to be additively manufactured as a single part. This monolithic implementation could be extended in future works to produce a compact dual-stack planar array design as in [29]. Monolithic designs of quasi-optical beamformers in additive manufacturing (AM) have already demonstrated reduced mass and improved performance by removing issues resulting from assembly tolerances [36]. Similar benefits are obtained with the proposed Rotman lens design.

The paper is organized as follows. Section 2 details the design of the double-ridged waveguide Rotman lens and associated linear array. Section 3 reports on the design adjustments required for additive manufacturing and the experimental validation. Finally, Section 4 provides some conclusions and perspectives for future works.

## 2. Double-Ridged Waveguide Rotman Lens Design in the K/K${}_{a}$-Band

**Figure 1:**
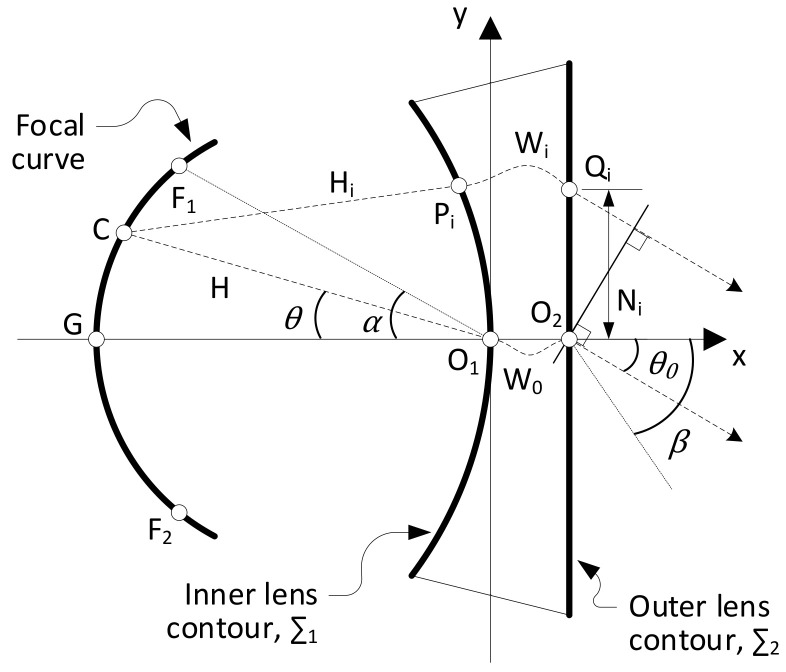
Rotman lens design parameters [20,37].

The Rotman lens is a particular case of planar (2-D) PPW-constrained lens beamformer suitable for linear array designs that produces three true focal points, one on-axis and two symmetrically arranged off-axis [20,24]. An adequate design of the focal arc enables us to reduce residual phase aberrations in between the focal points, resulting in a wide angular scanning range with stable radiation patterns. Figure 1 defines the main design parameters of a generalized form of Rotman lens [37], which introduces the scaling factor γ=sinβ/sinα, where α is the off-axis focal point angular position and β the corresponding beam pointing direction. The achievable scanning range is thus approximately ±β, as phase aberrations increase drastically beyond the off-axis focal points. In the original Rotman lens design, γ=1. The optimization of the focal arc is obtained by tuning the parameter $g={\mathrm{GO}}_{1}/F_{1}O_{1}$, corresponding to the ratio of the on-axis focal length over the off-axis focal length, *F*.

Here, the objective is to design a Rotman lens beamformer that covers both the downlink and the uplink frequency bands allocated to broadband satellite communications in the K/K${}_{a}$-band, 17.3–20.2 GHz, and 27.5–30 GHz, respectively. The detailed design of the Rotman lens is driven by the feeding port design, discussed in Section 2.1.

### 2.1. Feeding Port Design

**Figure 2:**
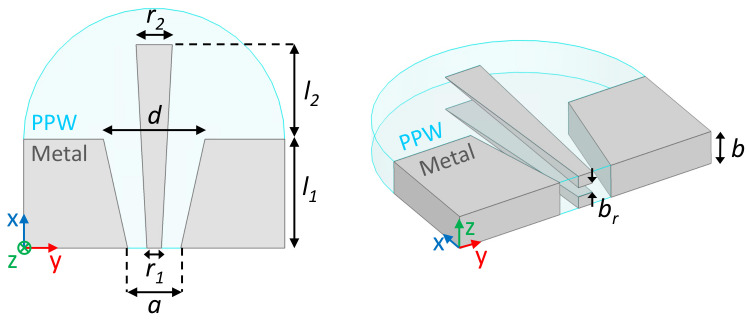
Double-ridged waveguide feeding port design parameters: top view (**left**) and isometric view (**right**).

A schematic of the double-ridged waveguide feeding port with associated parameters is provided in Figure 2. A double-ridged waveguide implementation is preferred as it leads to a shorter transition within the PPW for the same cutoff frequency when compared to a single ridge design. These port designs are of interest because they excite a quasi-TEM mode in the PPW section, providing true time delay operation within the lens cavity. They also enable smaller port-to-port spacing when compared to standard H-plane rectangular waveguides [33]. The design of the feeding port is similar to the one used in the Q-band Rotman lens design reported in [38], implementing a straight transition design here as this provided acceptable performance while simplifying the mechanical design. The feeding port has a constant height value, *b*, which is also the height of the PPW section.

The main parameter for the sizing of the Rotman lens is the aperture width of the feeding ports, *d*. Ideally, the value should be as small as possible, while ensuring good matching over the considered operating bandwidth. The starting point for the reference double-ridged waveguide was set to the WRD180 standard, as its nominal fundamental mode bandwidth (18–40 GHz) and cut-off frequency (15.12 GHz) are compatible with the targeted K/K${}_{a}$-band operation. The cross-section was slightly adjusted to improve the response, specifically in the lower part of the frequency range, using a full-wave model implemented with the commercial software Ansys HFSS. The corresponding design parameters are a=7.70mm, b=3.40mm, $r_{1}=1.92\;\mathrm{mm}$ and $b_{r}=0.98\;\mathrm{mm}$, where only *a* and $r_{1}$ have been adjusted compared to the WRD180 dimensions. The remaining optimized design parameters are d=8.86mm, $r_{2}=3.99\;\mathrm{mm}$, $l_{1}=9.66\;\mathrm{mm}$ and $l_{2}=13.55\;\mathrm{mm}$. The nominal feeding port design was optimized in an embedded configuration, including two adjacent ports on both sides, to reduce both reflections and port-to-port coupling with a goal of −20dB over the operating frequency bandwidth. Section 2.2 details the Rotman lens constructed from this feeding port design.

### 2.2. Rotman Lens Design

**Figure 3:**
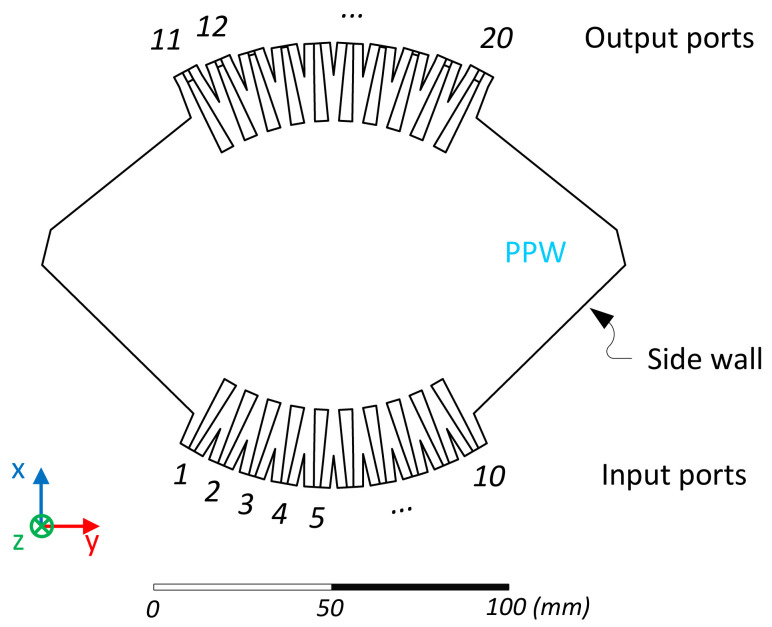
Full-wave model (Ansys HFSS) of the double-ridged 10 × 10 Rotman lens inner waveguide cavity, including transmission lines at the output ports.

A 10 × 10 Rotman lens was selected as a proof-of-concept, which required a trade-off of the main design parameters [24]. In particular, a compromise was required between frequency bandwidth and scanning range. Larger values of α led to degraded performance in the upper range of the targeted frequency band as a consequence of the larger incidence angle at the feeding ports. It was found that α=20° was acceptable for a K/K${}_{a}$-band design considering the size of the inner lens contour, $\Sigma_{1}$. Similarly, an optimal focal arc design leading to low theoretical phase aberrations typically requires g>1. However, this increases the curvature of the focal arc and degrades the performance when secondary effects, such as feeding port scattering, residual side wall reflections, etc., are taken into account. An acceptable trade-off was found for g=0.97. Considering the nominal feeding port aperture width reported above, the off-axis focal length was set to F=110mm or $F=11\lambda_{0}$, where $\lambda_{0}$ is the wavelength in free space at the highest operating frequency, 30 GHz. An internal Matlab tool was used to generate a step file combining the design parameters of the lens (Figure 1) with those of the feeding ports (Figure 2). The step file was then imported into Ansys HFSS to create the full-wave model. The shape of the side walls in the PPW cavity was adjusted to reduce residual reflections while implementing adequate absorbing boundary conditions [39]. The final full-wave model is illustrated in Figure 3.

**Figure 4:**
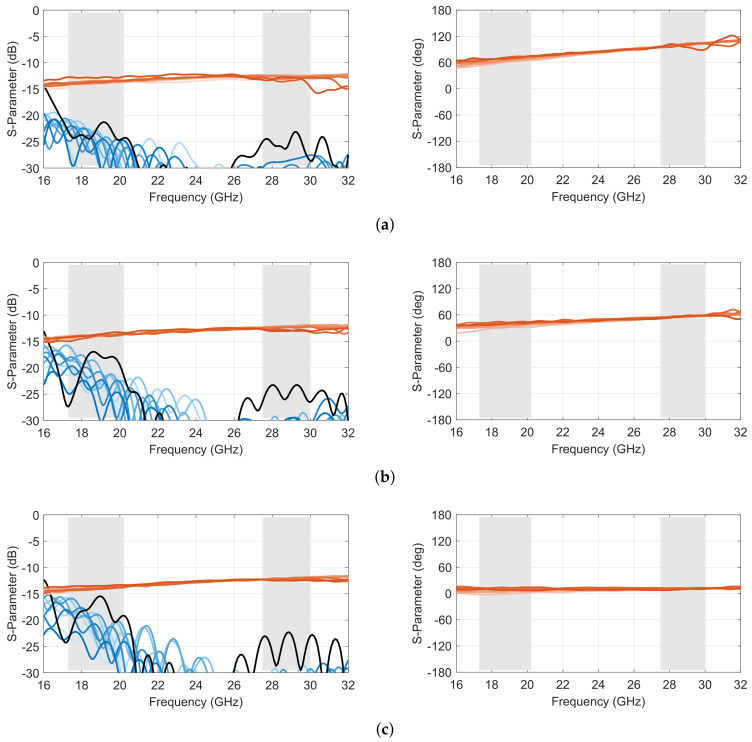
Simulated scattering parameters in amplitude (left) and adjacent port phase difference (right) of the double-ridged waveguide 10 × 10 Rotman lens design for (**a**) port 1, (**b**) port 3 and (**c**) port 5 (reflection coefficient in *black*; coupling coefficients in *shades of blue*; transmission coefficients in *shades of red*; operating frequency bands in *light grey*).

The scattering parameters of the full-wave model are reported in Figure 4, with port 1 corresponding to one of the outer ports (θ=α=20°), while port 5 is one of the inner ports (θ=4.44°), following the port numbering in Figure 3. These results are representative of the quasi-optical beamformer, with port 1 displaying some degradation in the transmission coefficients at the highest frequencies, while port 5 shows some degradation of the reflection and port-to-port coupling at the lowest frequencies. The results remained, nevertheless, acceptable for the targeted application, with a reflection and port-to-port coupling below –15 dB over the frequency bands of interest (light grey area in Figure 4), while the degradation on the transmission only affected port 11, corresponding to the extreme inner lens contour port on the same side as port 1.

**Figure 5:**
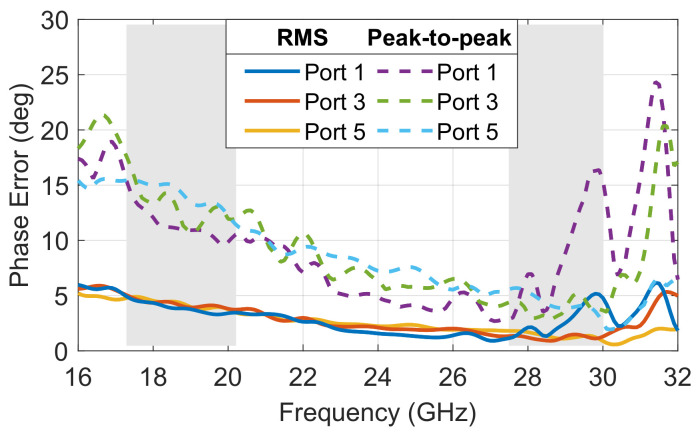
Simulated RMS and peak-to-peak phase error for selected ports of the double-ridged 10 × 10 Rotman lens design (operating frequency bands in *light grey*).

**Figure 6:**
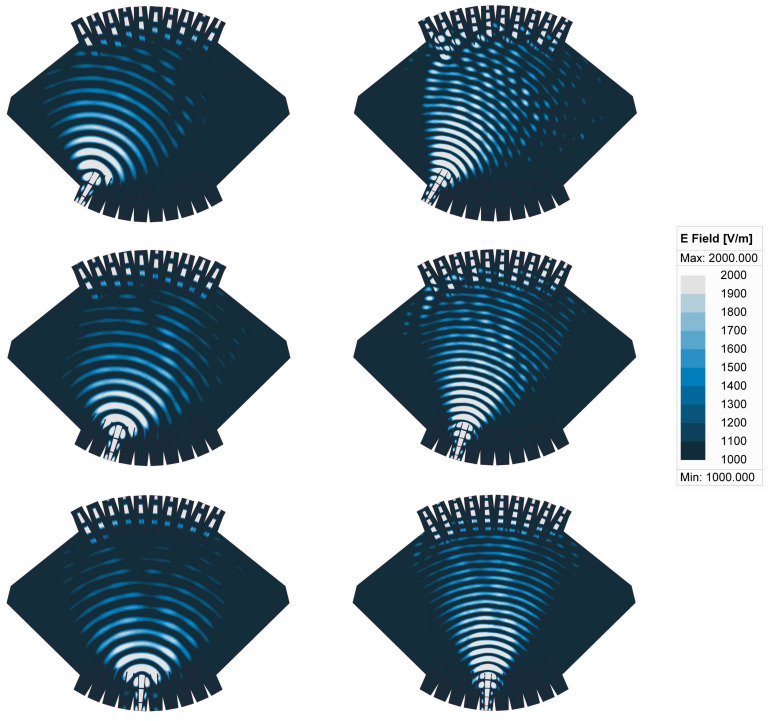
Simulated electric field distribution in the 10 × 10 Rotman lens design at 20 GHz (**left**) and 30 GHz (**right**) for port 1 (**top**), port 3 (**middle**) and port 5 (**bottom**).

The phase difference between adjacent ports also remained fairly constant at any frequency point across the band analyzed, providing the desired uniform phase progression, setting the beam pointing direction while having a phase slop characteristic of true-time delay beamformers. As expected, the phase difference between adjacent output ports increased with the distance of the input port considered to the center of the focal arc, with port 1 having the largest phase difference, while port 5 had a phase difference closer to zero producing a beam pointing near the boresight direction ($\theta_{0}=0{}^{\circ}$). The phase error, defined as the deviation from the average phase difference between adjacent output ports, is quantified as a function of frequency and the results are reported in Figure 5. The root mean square (RMS) error, corresponding to the standard deviation, remained below 5° across the operating bands, while the peak-to-peak error remained below 18°. The transmission line lengths, $W_{i}$ in Figure 1, were included in the model as double-ridged waveguides, which were more dispersive closer to the cut-off frequency. For this reason, the phase error was slightly worse at the lower frequencies. It is also noted that the phase error was fairly stable across the focal arc, confirming the excellent stability of the Rotman lens across the scanning range. Port 1, which is one of the off-axis focal points, has phase errors similar to those of the other ports, although slightly worse in the upper band, confirming the phase errors are dominated by implementation aspects such as transitions and reflections within the cavity not accounted for in the simplified ray-tracing model of the Rotman lens [20,38]. Overall, it is noted that the operation could be extended with acceptable performance up to 31 GHz to include the governmental band allocated to broadband satellite communications, confirming the very wide band operation of the beamformer. The electric field distribution obtained with the full-wave model at 20 and 30 GHz are reported for ports 1, 3 and 5 in Figure 6.

To extend slightly the scanning range while avoiding grating lobes in the visible domain, the scaling factor was set to γ≈1.33, such that the 10 beams produced cover a range of 27° with a beam-to-beam angular spacing of 6°. The linear array considered has an element spacing of 6.28 mm, corresponding to 0.63 $\lambda_{0}$ at 30 GHz. Here, we focus on the quasi-optical beamformer design and assume the output ports are connected via equal-length coaxial cables to the elements of the linear array using adequate transitions. Section 3 discusses the Rotman lens design adaptation to additive manufacturing and associated experimental results.

## 3. Experimental Verification of the Monolithic Rotman Lens Design

### 3.1. Design for Manufacture Considerations

For the prototype reported here, laser powder bed fusion (LPBF) AM was chosen [40]. LPBF involves the sequential deposition of metal powder layers, AlSi10Mg aluminum alloy in this case, with each layer having a height range of approximately 20–30 µm. The choice of material was driven here by the targeted space applications, with antenna systems often exposed to the harsh space environment, thus requiring components providing a good compromise between electrical and mechanical properties [40]. The uniform deposition of the metal powder on the printing bed is accomplished using a recoater. Selective melting of the metal powder is achieved using an energy source, a laser in this case. After melting the metal powder layer, a uniform layer is added again by the recoater, and the melting process is repeated until the final part is generated.

**Figure 7:**
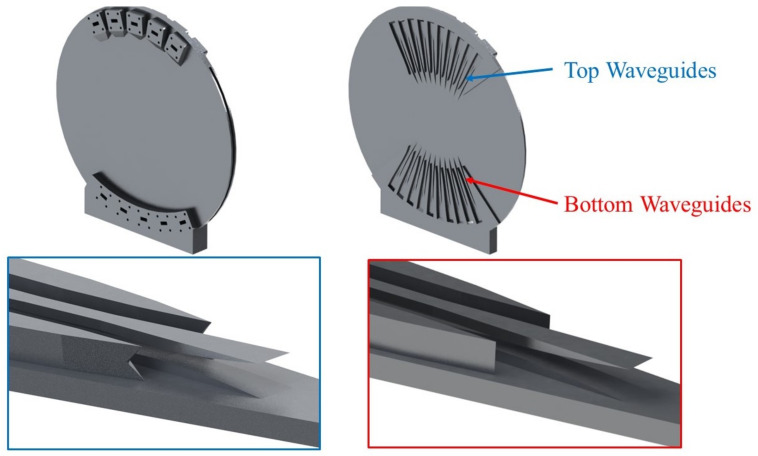
Close-up view of the top (**left**) and bottom (**right**) feeding waveguides in the monolithic 10 × 10 Rotman lens prototype.

While AM technologies provide some flexibility in the RF design by removing mechanical interfaces and simplifying the overall assembly, they do introduce some printing constraints, which require careful consideration [40]. The most important parameter to be defined is the printing direction, expected to provide a stable PPW thickness without requiring excessive supporting structures. It has been decided to print along the central focal axis of the lens (x-axis in Figure 1). This requires redesigning the ports on the top side of the PPW section to avoid overhangs and underhangs. To this end, starting from the feeding port design in Figure 2, an adaptation was made to introduce a roof with an inverted triangular shape, with angles at 45°, compatible with the constraints of AM. The details of this design adaptation are highlighted in Figure 7. For what concerns dummy ports, the redesign effort would be quite significant with questionable impact on performance due to their orientation with respect to the printing direction. For this reason, it was decided to keep the sides of the PPW section open so absorbing material may be inserted in the cavity. For that purpose, ABS-MLSE samples from ABS Technics BV are used. ABS-MLSE is a thin and flexible silicon absorber with magnetic loading, providing a typical attenuation higher than 100 dB/cm in the K-band. The material is cut to produce a cavity shape in line with the full-wave model of Figure 3.

The Rotman lens prototype also required some adaptation for test purposes. To enable the use of available standard waveguide calibration kits, transitions from a double-ridged waveguide to a rectangular waveguide were added. The prototype was completed with alternating E-plane bends that enable inserting WR42 flanges without significantly increasing the length of the transitions. These require the addition of supporting structures in the final part of the waveguides, which are removed by drilling, as a part of the post-processing stage also required for the flanges. Moreover, the manufactured part was subjected to a sandblasting process, with glass micro-spheres, to uniformly reduce the surface roughness, Ra, to 4–6 µm.

**Figure 8:**
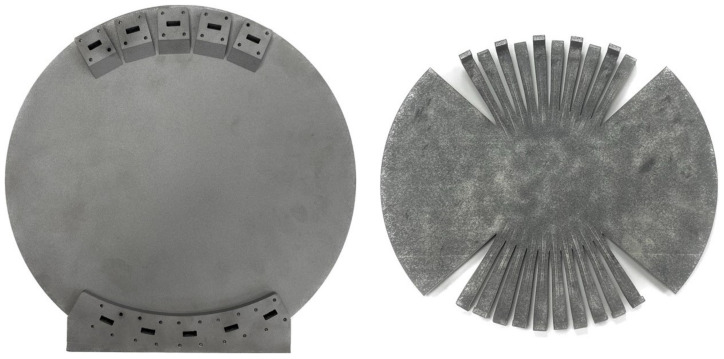
Photograph of the manufactured monolithic 10 × 10 Rotman lens prototype (**left**) and inner cavity prototype (**right**).

The manufactured prototype is shown in Figure 8. A prototype of the inner cavity, also shown in Figure 8, has been manufactured and facilitates the visualization of the final RF design, in particular, the alternating E-plane bend arrangement to accommodate the WR42 flanges. The multi jet fusion (MJF) AM technique was chosen in this case. MJF is a type of AM technology that uses a combination of inkjet printing and powder bed fusion. The process involves depositing a thin layer of powder material, usually a type of nylon, on a build platform. Then, using an inkjet array, a liquid agent is selectively applied to the powder layer, fusing the particles together in the desired pattern. The process is repeated, with successive layers of powder and inkjet-fused material, until the final object is formed.

### 3.2. Experimental Results

**Figure 9:**
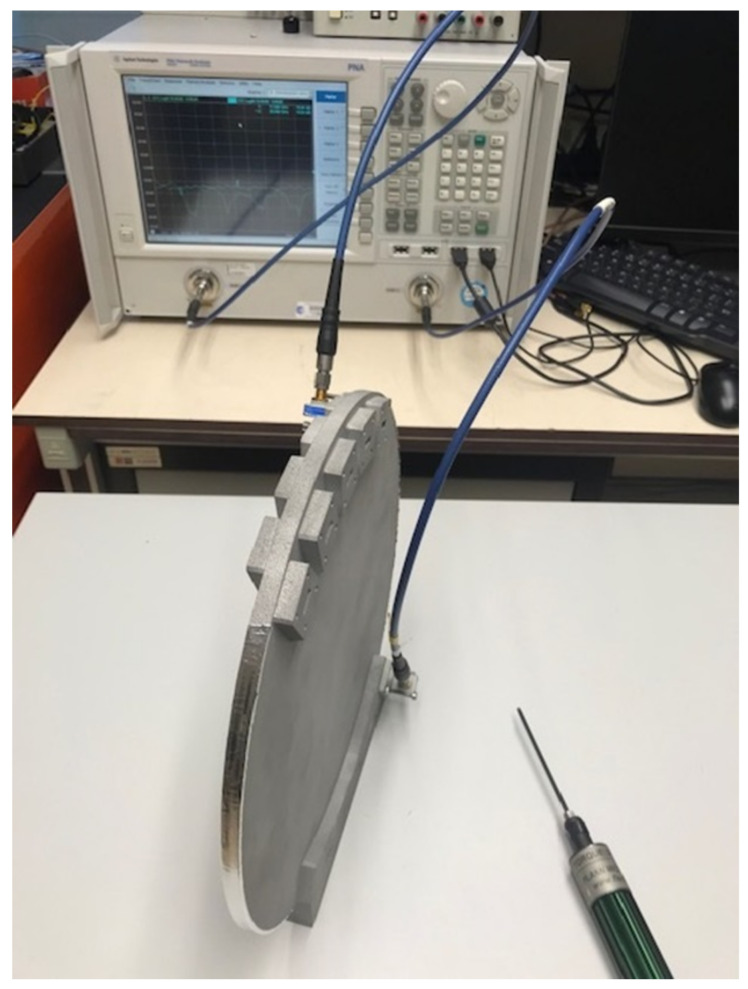
Test setup for the measurement of the scattering parameters of the 10 × 10 Rotman lens prototype.

To simplify the overall test setup and test sequence, it was decided to keep unused waveguide ports open. Thanks to the low port-to-port coupling, this is found to have a moderate impact on the performance and still enables the validation of the concept. The measurements were performed in the Microwave Laboratory at the European Space Research and Technology Center (ESTEC), Noordwijk, The Netherlands. The test setup is shown in Figure 9. A WR42 calibration kit was used to perform scattering parameter measurements over the frequency range 16–24 GHz, while custom waveguide transitions and a WR34 calibration kit were used to measure over the range 24–32 GHz. The WR42-to-WR34 transitions were also measured back-to-back to evaluate their impact on insertion losses in the upper frequency range, with a measured mean value around 0.11 dB.

**Figure 10:**
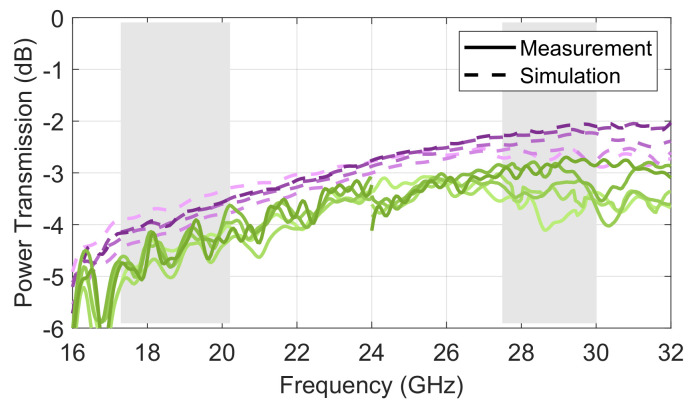
Power transmission in the double-ridged 10 × 10 Rotman lens prototype for port 1 to 5, from lighter to darker color (measurements in *shades of green*; simulations in *shades of purple*).

First, the power transmission, including spillover, mismatch and insertion losses, are reported in Figure 10. On absolute values, the losses are higher in the lower band than in the higher band. This is mostly because of the higher spillover losses, as the H-plane pattern of the feeding ports has a wider beamwidth at the lower frequencies. It is also noted that the oscillations are larger in the lower band. This is predominantly due to the increased scattering coming from the higher spillover. As anticipated, the insertion losses, which may be evaluated as the difference in power transmission between the ideal model (i.e., perfect electric conductor boundary condition) and the measured prototype, increase with frequency. The difference, on average, was around 0.5 dB in the lower band and increased to 0.8 dB in the upper band. It is noted, however, that a significant portion of the electrical path is due to the transitions for test purposes. The actual insertion losses of the Rotman lens core design are estimated to be about half of the measured figures, as the waveguide transitions have a combined length of about 120 mm, while the average path in the core design is around 150 mm. It is also noted that the measured results show some discontinuity at 24 GHz. This is a consequence of differential calibration and measurement uncertainties between the two calibration kits used for these tests. The results are nevertheless in line with expectations within the operating bands of interest.

**Figure 11:**
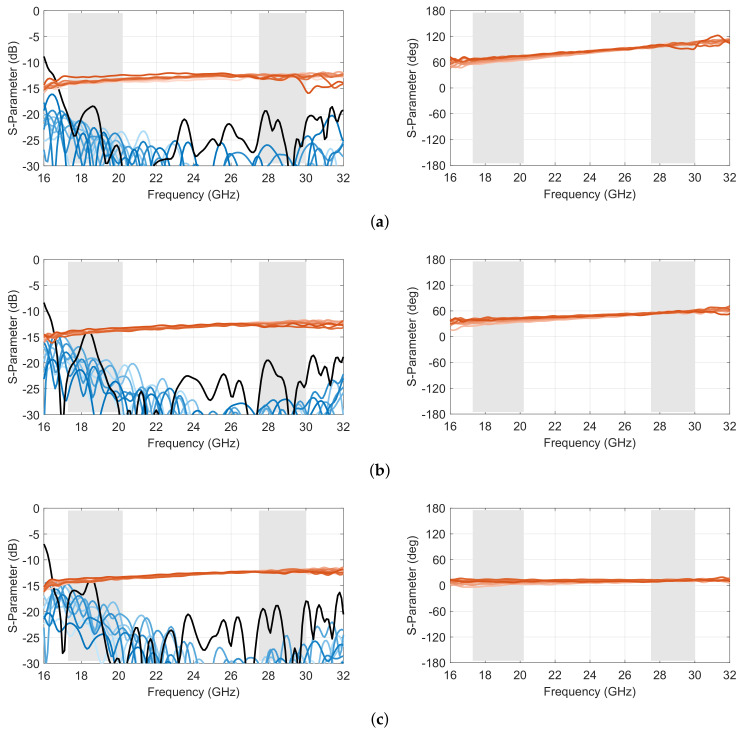
Simulated scattering parameters in amplitude (**left**) and adjacent port phase difference (**right**) of the double-ridged waveguide 10 × 10 Rotman lens prototype for (**a**) port 1, (**b**) port 3 and (**c**) port 5 (reflection coefficient in *black*; coupling coefficients in *shades of blue*; transmission coefficients in *shades of red*; operating frequency bands in *light grey*).

**Figure 12:**
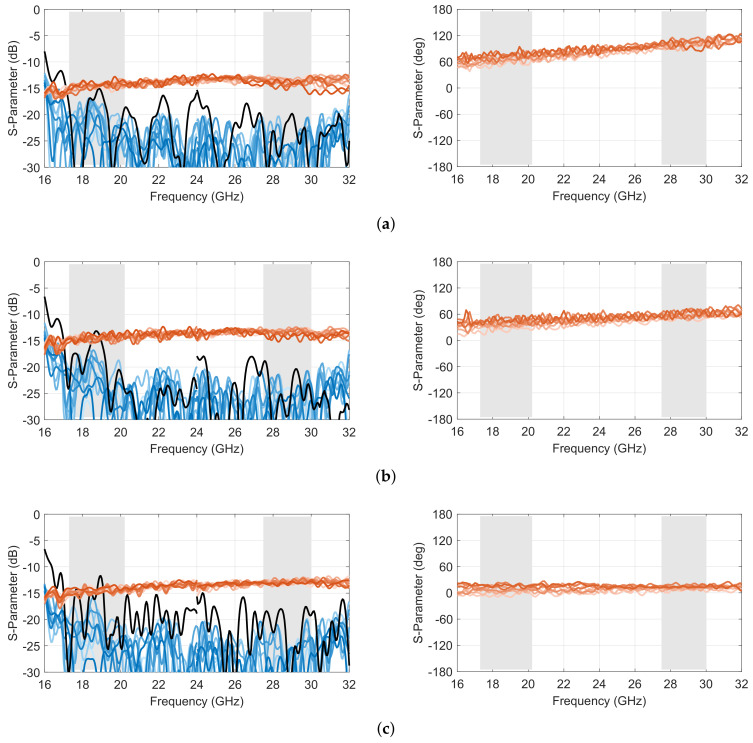
Measured scattering parameters in amplitude (**left**) and adjacent port phase difference (**right**) of the double-ridged waveguide 10 × 10 Rotman lens prototype for (**a**) port 1, (**b**) port 3 and (**c**) port 5 (reflection coefficient in *black*; coupling coefficients in *shades of blue*; transmission coefficients in *shades of red*; operating frequency bands in *light grey*).

A representative selection of scattering parameters of the complete 10 × 10 Rotman lens prototype is reported in Figure 11 and Figure 12, corresponding to simulated and measured results, respectively. Compared to the core design reported in Section 2, this complete prototype, including transitions, shows slightly degraded reflection and isolation coefficients, with a worst case of −13.6 dB in the lower band in simulation and −11.5 dB in measurements. Both worst-case results are obtained for port 5, which corresponds to one of the inner ports. This is because the scattering from the inner lens contour tends to reflect back toward the center of the focal arc due to its shape, while this reflection follows a more specular direction for offset input ports. The results remain nevertheless acceptable. The level of the transmission coefficients is also fairly in line with predictions, with slightly higher oscillations due to secondary effects not accounted for in the model and the test configuration (i.e., open-ended waveguides). Similar oscillations are also observed in the phase response as a consequence of the test setup. However, the phase variations by frequency remained fairly in line with predictions.

**Figure 13:**
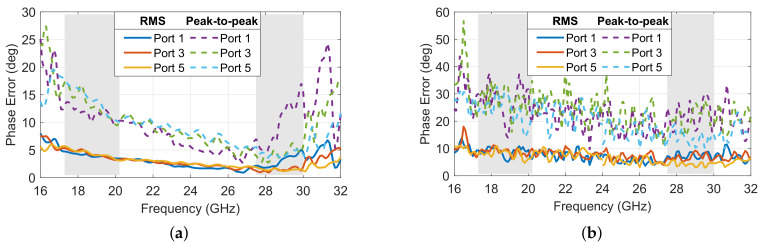
(**a**) Simulated and (**b**) measured RMS and peak-to-peak phase error for selected ports of the double-ridged 10 × 10 Rotman lens design (operating frequency bands in *light grey*).

The phase error versus frequency was evaluated in simulation and measurement for selected ports, and the results are reported in Figure 13. The simulated results are very similar to those reported in Figure 5, as the transitions included in all ports have no impact on the phase difference. The measured phased error is about twice the predicted one, with a standard deviation below 10° and a peak-to-peak error below 38°. Variations of up to 10° in peal-to-peak error were observed across the operating band, while the RMS values were more stable versus frequency. As expected from simulations, the phase error is slightly worse in the lower frequency band. The values remain nevertheless acceptable considering the test setup. The accurate characterization of such large beamformers may benefit from automation, which will be considered in future works.

**Figure 14:**
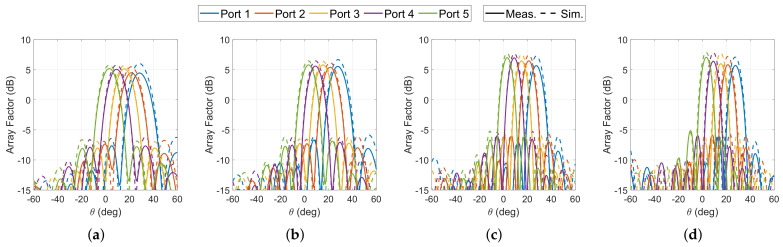
Synthesized array factors obtained with the scattering parameters of the double-ridged waveguide 10 × 10 Rotman lens prototype at (**a**) 17.3 GHz, (**b**) 20.2 GHz, (**c**) 27.5 GHz and (**d**) 30 GHz, assuming an array spacing of 6.28 mm (measurement in *solid lines*; simulation in *dashed lines*).

Finally, synthesized array factors obtained with the scattering parameters of the 10 × 10 Rotman lens prototype are reported in Figure 14, assuming a linear array with a spacing of 6.28 mm as discussed in Section 2.2. Numerical results are reported for the frequencies at the edge of the bands considered, i.e., 17.3 GHz, 20.2 GHz, 27.5 GHz and 30 GHz. The results are not normalized to report the losses with reference to the ideal 10-element array factor adding 10 dB to the elementary pattern gain. The losses obtained were slightly higher than those reported in Figure 10 because phase errors also translate into a maximum gain degradation. The average difference in peak array factor between simulation and measurement was 0.9 dB in the lower frequency band and 1.2 dB in the upper band. The difference typically tends to increase toward the outer ports. For example, at 30 GHz, the difference was only 0.8 dB for port 5, but 1.4 dB for port 1, the outermost feeding port. These results are in line with previously reported numerical results [38] indicating that phase aberrations increase with the pointing angle despite the presence of the off-axis focal point. This is because secondary effects not accounted for in the simplified ray-tracing models become predominant. Nevertheless, the results reported confirm the good stability of the patterns across the wide frequency band analyzed, both in terms of pointing direction and side lobe levels, confirming the potential of this design for wide-band and dual-band applications in the K/K${}_{a}$-Band.

## 4. Conclusions

This paper has provided the first experimental validation of a fully-metallic Rotman lens design manufactured in a single block. The use of additive manufacturing enabled the removal of all the screws, avoiding potential misalignments during assembly. A thorough characterization was conducted with a design operating in the K/K${}_{a}$-band and key results were reported. Overall, the performance of the prototype is well in line with the predictions of the full-wave model. Measured losses, ranging from 0.5 dB to about 0.8 dB, on average, are in line with expectations for the implemented manufacturing process, and are, in part, due to the long transitions required for test purposes. The actual losses of the core design are about half these figures. The synthesized array factors demonstrate very stable beamforming across the wide frequency range analyzed. Future works will address the feasibility of a compact dual-stack configuration for planar array design.

## Data Availability

The data reported in this paper is available from the corresponding author, upon reasonable request.
